# Cell Pathology

**Published:** 1865-12

**Authors:** 


### CELL PATHOLOGY.

Dr. Bennett stated that a cell-pathology had naturally sprung from the cell-theory, as originally framed by its founders, Schleiden and Schwann, which had greatly extended the boundaries of medical science. The cell pathology of Virchow, however, was based upon a law he sought to establish, viz., that every cell sprang from a pre-existing cell, and that we must not transfer the seat of oval action to any point beyond the cell. This supposed law, he maintained, was opposed by so many histological facts as to be altogether untenable. He begged especially to draw attention to the origin of puss-cells, which Virchow and some of his followers had represented as originating in the interior of connective tissue corpuscles. Dr. Bennett and his pupils had frequently sought, by passing setons through the skin and muscles of animals, to observe in the inflamed tissues the appearances which had been figured in support of Virchow's views, but had never succeeded in seeing pus-cells within pre-existing cells. Henle had pointed out that the error had originated in mistaking the triangular spaces observable, on a transverse section, between the bundles of various fibrous tissues, for cells; as in these, unquestionably, puss was very likely to collect. Dr. Bennett further believed that the tendency of many cells to enlarge as the result of irritation, and to multiply themselves endogenously, as shown by himself, by Roberts, Goodsir, Redfern, and other pathologists, was another source of mistake among the younger histologists. The granules and included cells so formed were mistaken by them for those of pus, though easily separated from them. He called attention to a series of preparations (which were exhibited,) showing suppuration in inflamed eye-balls, and in pneumonic lungs, in which pus-cells might be seen in all stages of formation—originating from a coagulated molecular exudation, unconnected with any pre-existing cells whatever. In the sections of lung more especially, the fibrous tissue of the organ surrounding the air-cells might be seen to be quite healthy. In the coagulated exudation, on the other hand, the molecules might be observed at first uniformly filling up the air-vesicle; then formed into masses, varying in size from the twenty-thousandth to the one-thousandth of an inch in diameter. The latter were sounded, and were identical with pus-corpuscles. He believed that these bodies, therefore, were formed by an aggregation of smaller particles or molecules, composed originally of the coagulated exudation. It was certain that, in the situations referred to, they did not originate in pre-existing cells, as no such cells could be seen. If, as might be supposed, they sprang from the epithelial cells lining the chambers of the eye or the air-vesicles, such cells would be seen, enlarged, and containing the pus-bodies. But his preparations and numerous examinations of the part when diseased had proved to him that no such cells were mixed with the exudation, or in any connected with the formation of pus.—*British Medical Journal.*

